# Real-World Assessment of the Xpert MTB/XDR for Detecting Isoniazid and Second-Line Drug Resistance Among TB Patients

**DOI:** 10.3390/ijms27062597

**Published:** 2026-03-12

**Authors:** Andrei Makhon, Sivan Fuchs, Mor Rubinstein, Maya Brodsky, Zeev Dveyrin, Noa Tejman-Yarden, Yelena Losev

**Affiliations:** 1 National Public Health Laboratory, Public Health Directorate, Ministry of Health, Tel Aviv 6810416, Israel; andrei.makhon@phlta.health.gov.il (A.M.); sivan.fuchs@phlta.health.gov.il (S.F.); mor.rub@phlta.health.gov.il (M.R.); zeev.dveyrin@phlta.health.gov.il (Z.D.); noa.tejman-y@moh.gov.il (N.T.-Y.); 2 Clalit Health Services, Regional Laboratory of Haifa and Western Galilee, Nesher 3688847, Israel; mayabr1@clalit.org.il

**Keywords:** *Mycobacterium tuberculosis* complex (MTBC), whole genome sequencing (WGS), drug resistant TB (DR-TB), extensively drug resistant TB (XDR-TB), Xpert^®^ MTB/XDR (GXXDR)

## Abstract

Rapid and accurate detection of drug-resistant tuberculosis (DR-TB) is critical for effective treatment and containment. The Xpert^®^ MTB/XDR (GXXDR) assay is designed to detect *Mycobacterium tuberculosis* complex (MTBC) and resistance to isoniazid and second-line anti-TB drugs directly from clinical specimens. We evaluated the clinical performance of GXXDR using 61 MTBC-positive specimens with available phenotypic drug susceptibility testing results. GXXDR results were compared to a phenotypic drug susceptibility test (pDST) and whole-genome sequencing (WGS) to assess sensitivity, specificity, and concordance. Resistance to isoniazid, fluoroquinolones, amikacin, capreomycin, and ethionamide was analyzed. Sensitivity comparisons between GXXDR, WGS, pDST, and manufacturer data were performed using Fisher’s exact and Tango tests. GXXDR demonstrated a high specificity for most drugs and a strong sensitivity for isoniazid (93.8%) and fluoroquinolone (92.3%), consistent with manufacturer reports. In contrast, the sensitivity for amikacin (58.3%), capreomycin (35.7%), and ethionamide (27.3%) was significantly lower than stated by the manufacturer (91.9%, 84.0% and 64.7%, respectively), likely due to resistance mutations outside the assay’s target regions. Sensitivity concordance of GXXDR with WGS was high for all drugs, except ethionamide. The GXXDR assay enables rapid and reliable detection of isoniazid and fluoroquinolone resistance in clinical settings, though sensitivity for certain second-line drugs may be affected by regional genetic diversity. These findings underscore the importance of integrating local epidemiological data to optimize molecular diagnostics for DR-TB.

## 1. Introduction

Tuberculosis (TB), caused by *Mycobacterium tuberculosis* (MTB), is the leading cause of death from a single infectious disease [1]. In 2023, an estimated 8.2 million people were newly diagnosed with TB worldwide, with a global incidence of 134 cases per 100,000 individuals [2]. In contrast, Israel has a low TB burden, with 196 reported cases in 2024, corresponding to an incidence rate of two cases per 100,000. Most TB patients in Israel are individuals born in TB-endemic countries, including migrant workers and Jewish immigrants [3].

Drug-resistant TB (DR-TB) remains a major global health challenge. The World Health Organization (WHO) classifies TB drug resistance into five categories: isoniazid-resistant TB; rifampicin-resistant TB (RR-TB); multidrug-resistant TB (MDR-TB), which is defined as resistance to both isoniazid (INH) and rifampicin (RIF); pre-extensively drug-resistant TB (pre-XDR-TB), defined as MDR-TB/RR-TB with an additional resistance to any fluoroquinolone (FQ); and extensively drug-resistant TB (XDR-TB), defined as pre-XDR-TB with an additional resistance to at least one of either bedaquiline or linezolid [2].

The successful treatment of TB, including drug-resistant forms, depends on combination therapy administered over variable treatment durations. Current guidelines recommend a 4-month regimen consisting of INH, RIF, moxifloxacin (MFX), and pyrazinamide for the treatment of drug-sensitive tuberculosis. For patients with MDR-TB, a 6-month regimen comprising bedaquiline, pretomanid, linezolid, and MFX (BPaLM) is currently recommended. Additional agents used in TB treatment include aminoglycosides, such as amikacin (AMK) and capreomycin (CAP), ethambutol, streptomycin (STR), and ethionamide (ETH) [4].

Rapid detection of TB infection and antibiotic resistance is key to effective treatment and containment of outbreaks. Conventional diagnostic tests include sputum smear microscopy and a culture using liquid and solid media. Although these tests are still widely used, they have important drawbacks. Sputum smear microscopy has a detection limit of 5000–10,000 bacilli per milliliter of sputum and cannot identify antibiotic resistance. Culture, while considered the gold standard for confirming TB and performing phenotypic drug susceptibility testing (pDST), requires several weeks to produce results, is labor-intensive, and poses a serious biohazard risk to laboratory workers [5].

Another approach for detecting antibiotic resistance is whole-genome sequencing (WGS). In 2023, the WHO published the second edition of its catalog of all known mutations in *Mycobacterium tuberculosis* complex (MTBC) and their association with drug resistance, which includes approximately 30,000 variants linked to resistance against 13 drugs [6]. Although WGS-based drug susceptibility testing is effective and faster than conventional phenotypic testing, it is not yet widely implemented in low- and middle-income countries due to its high cost [7]. Additionally, it is now primarily performed on cultures rather than directly on samples, which is also time-consuming.

WHO recommends a set of technologies for the detection of TB and DR-TB, which are organized into two categories: initial tests for TB diagnosis and follow-on diagnostic tests performed after TB confirmation. The first category includes Truenat assays, Xpert MTB/RIF and its successor, Xpert MTB/RIF Ultra [8,9]. These assays are rapid, easy to use, and fully automated real-time polymerase chain reaction (PCR) tests for detecting MTBC and RIF resistance. However, they cannot detect INH or second-line anti-TB drug resistance. Another group of initial tests consists of automated, moderate-complexity nucleic acid amplification tests (NAATs), which are able to detect not only MTBC and RIF resistance but also INH resistance [5].

Follow-on diagnostic tests include low-complexity automated NAATs, line probe assays, high-complexity reverse hybridization NAATs, and targeted NGS tests. The first-in-class product for low-complexity automated NAATs for detecting resistance to INH and second-line anti-TB drugs is the Xpert MTB/XDR assay (GXXDR) [5]. When it was performed on the GeneXpert Instrument Systems, this qualitative nested real-time PCR in vitro diagnostic test detects XDR-MTBC DNA in unprocessed sputum samples, concentrated sputum sediments, or culture isolates. MTBC detection is based on the single-copy *inhA* promoter target. The resistance to INH is identified through mutations in the *inhA* promoter region and the *katG*, *fabG1*, and *oxyR–ahpC* intergenic region. FQ resistance is detected via mutations in *gyrA* and *gyrB*; ethionamide resistance through the *inhA* promoter; and resistance to AMK, kanamycin (KAN), and capreomycin (CAP) via the *rrs* gene and *eis* promoter [10].

The assessment of the sensitivity, specificity, and concordance of the GXXDR with WGS and phenotypic assays is of critical importance, particularly in light of regional variants, sample quality, and the distinctive laboratory conditions present in the National Mycobacterium Reference Laboratory (NMRL) of Israel, all of which may impact the assay’s performance.

This study evaluates the diagnostic sensitivity and specificity of the GXXDR assay in a real-world scenario as an INH and second-line drug assessment tool when compared with the gold-standard pDST and examines its performance relative to WGS, using 64 tuberculosis specimens and cultures. The assay’s accuracy and sensitivity were calculated and compared with the manufacturer’s data to verify its appropriateness for implementation within the local setting.

The evaluation of the GXXDR assay’s performance in Israel will facilitate evidence-based decision-making regarding its utilization for the rapid detection of drug resistance in TB. The outcomes of this study are anticipated to advance patient management and contribute to the containment of resistant bacterial strains.

## 2. Results

A total of 64 MTB positive samples with available pDST and GXXDR test results were included in this study. One sample was identified as a mixed culture of MTB and *M. simiae*, and two samples were contaminated. These three samples were excluded from further analysis, leaving 61 samples for evaluation. The pDST detected resistance to INH in 32 of the 61 (52%) tested samples, to FQ in 13 of the 44 (30%) tested samples, to AMK in 12 of the 45 (27%) tested samples, and to CAP in 14 of the 36 (39%) tested samples, and out of 45 samples tested to ETH resistance, 22 were resistant (49%).

GXXDR demonstrated a high accuracy for INH and FQ (96.7% and 97.7%, respectively), whereas a lower accuracy was observed for AMK, CAP, and ETH (86.7%, 75.0%, and 64.4%, respectively). The precision was 100% for all drugs except AMK (87.5%).

**Figure 1:**
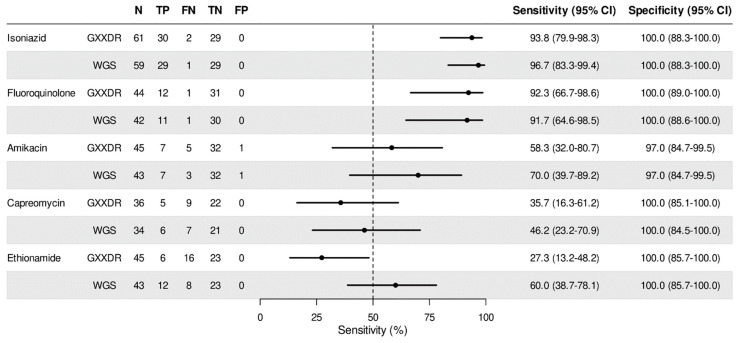
Sensitivity and specificity of the GXXDR and WGS compared with pDST. N—Number of samples. TN—true negatives. TP—true positives. FN—false negatives, FP—false positives.

GXXDR demonstrated 100% specificity for most antibiotics except for AMK (Figure 1, Supplementary Table S1), which had one false-positive result. These findings are consistent with the manufacturer’s reported specificity of 98–99% for all drugs. Sensitivity results for INH (93.8%) and FQ (92.3%) were comparable to the manufacturer’s data (*p* = 1). In contrast, AMK, CAP, and ETH showed significantly lower sensitivities than reported. For AMK, the sensitivity in the current study is 58.3% vs. the 91.9% that was reported by the manufacturer (*p* = 0.0058). For CAP, the sensitivity is 35.7% vs. the 84.0% that was reported by the manufacturer (*p* = 0.0041), and for ETH, the sensitivity is 27.3% vs. the 64.7% (*p* = 0.0017) reported by the manufacturer.

GXXDR showed comparable diagnostic accuracy to WGS, with identical results in detecting FQ resistance. The two methods disagreed only in one sample for INH resistance and in an additional sample in both AMK and CAP, with WGS correctly identifying resistance in both samples. Only for ETH, WGS demonstrated statistically significantly higher diagnostic accuracy (*p* = 0.0082), correctly identifying resistance in six additional samples compared to GXXDR.

## 3. Discussion

In this study, we systematically evaluated the sensitivity and specificity of the GXXDR assay for detecting drug resistance in MTB, using pDST as the reference standard. Although GXXDR is increasingly implemented in routine diagnostic workflows due to its rapid processing time and ease of use, its accuracy for certain resistance-conferring mutations remains a subject of debate. Alongside pDST, we used WGS to capture a more comprehensive profile of resistance-conferring mutations. By directly comparing clinical sensitivity and specificity from both platforms across the same set of clinical isolates, we provide an updated real-world evaluation of assay performance.

Anti-TB drugs target essential bacterial processes, but resistance arises through distinct molecular mechanisms. INH is a prodrug that is activated by the mycobacterial catalase–peroxidase enzyme KatG, leading to the formation of reactive intermediates that inhibit mycolic acid biosynthesis, which is a critical component of the mycobacterial cell wall. InhA, which is targeted by both INH and ETH, functions as an enzyme in the fatty acid synthesis pathway. Mutations in the *katG*, which affect INH activation, are known to confer resistance to INH, as well as mutations in the promoter of *inhA*, which lead to its over-expression, thus compensating for the loss of this enzyme’s activity caused by the drug [11,12].

Clinically, high-level resistance to INH has been associated with mutations at codon 315 of the *katG* gene, as well as mutations in the regions upstream of the *inhA* promoter, including the *fabG1* gene and the *oxyR–ahpC* intergenic region. In contrast, specific mutations within the *inhA* promoter region alone are typically associated with low-level INH resistance [13].

FQs are broad-spectrum antibacterial agents that act by increasing DNA strand breaks mediated by type II topoisomerases, such as gyrase and topoisomerase IV. MTB is unique in encoding only DNA gyrase, which is a heterotetramer composed of two subunits, GyrA and GyrB. Clinically relevant mutations in the quinolone resistance-determining region in the genes *gyrA* and *gyrB* confer FQ resistance by disrupting the enzyme–drug interaction and reducing FQ binding affinity. Depending on the degree of interference with the drug binding, some mutations, such as *gyrA* A90V and D94A, confer low-level resistance, while others, like *gyrA* G88Cs, D94N, D94G, D94H or D94Y, confer high-level resistance. The combination of several low-level resistance mutations has a synergetic effect and is considered high-level resistance [13,14,15,16].

Overall, our results show that GXXDR demonstrates high accuracy for detecting resistance to INH and FQ, both of which are predominantly driven by well-characterized mutations in canonical resistance loci. The GXXDR assay yielded only two false-negative INH results. In one isolate, this discrepancy was explained by a *katG* 45_46insT, a mutation not targeted by the assay (Supplementary Table S2). In the second isolate, WGS identified two mutations categorized as uncertain significance, *katG* 412T>C and *glpK* 572_573insC, inside the *glpK*’s homopolymeric tract of seven cytosines. It is important to mention that frameshift mutations in the 7C homopolymeric tract were shown to increase drug tolerance in a rapidly reversible manner, resulting in phase variation. Because *glpK* has a key role in glycerol metabolism, such mutants are characterized by slow growth and induction of stress response, rendering the bacteria antibiotic-tolerant [17]. For FQ resistance, both WGS and GXXDR failed to detect resistance in a single isolate that carried three mutations (*gyrA* 863C>A, Rv1129c 404T>G, and Rv2752c 1664A>G) in separate genes categorized as of uncertain significance.

Aminoglycosides are a class of antibiotics that inhibit protein synthesis. This group includes STR, AMK, KAN, and CAP, the latter being a cyclic peptide. KAN and AMK inhibit protein synthesis by targeting the 16S rRNA (encoded by the *rrs* gene) of the 30S ribosomal subunit. One of the most common mutations, 1401A>G in the *rrs* gene, confers cross-resistance to AMK, KAN, and CAP, although cross-resistance between KAN and AMK is not complete. Low-level resistance to KAN has been linked to mutations in the promoter region of the *eis* gene, which encodes an aminoglycoside acetyltransferase. Mutations at the −10 and −35 positions of the *eis* promoter result in protein overexpression and low-level resistance to KAN but not AMK, whereas a mutation at position −14 confers resistance to both KAN and AMK.

CAP binds at the interface of the small and large ribosomal subunits. Resistance to CAP has been associated with mutations in the *tlyA* gene, which encodes 16S/23S rRNA (Cytidine-2′-O)-methyltransferase. Mutations in *tlyA* prevent methylation of rRNA, reducing CAP binding efficiency and leading to antibiotic resistance [13,18,19].

The GXXDR test exhibited reduced sensitivity in detecting AMK, CAP and ETH resistance. Resistance to these drugs is caused by a diverse mutational landscape or mutations located outside the assay’s target regions. Notably, one isolate with a false-negative GXXDR result for both AMK and CAP carried the well-characterized 1401A>G mutation with a frequency of 0.96 in the 16S rRNA gene, which is a canonical marker of high-level resistance. It remains unclear why this mutation was not detected by the GXXDR assay, suggesting either a technical limitation or an isolated assay failure that warrants further investigation. Because the mutant allele was present at such a high frequency, the likelihood of a mixed culture contributing to this discrepancy is improbable. Another example of a GXXDR false-negative result is an isolate carrying a 10bp loss-of-function deletion in the *tlyA* gene, which is a well-established cause of CAP resistance that lies outside the assay’s target range, and, therefore, cannot be detected by GXXDR. In general, within the GXXDR assay, detection of aminoglycoside resistance is limited to the mutations located in the *rrs* and *eis* gene regions. The low sensitivity for ETH can be explained by the limited target regions included in the GXXDR assay for this drug. As a result, six isolates harboring resistance-conferring mutations in the *ethA* gene were incorrectly classified as susceptible due to the fact that the GXXDR assay does not target this gene. Furthermore, one of these isolates carried the –154G>A mutation in the *inhA* promoter, which also lies outside the GXXDR assay’s detection region [10].

GXXDR showed a high specificity for all drugs, having only one false-positive result in AMK testing, which was an isolate carrying the *eis* –14C>T promoter mutation that exhibited a borderline AMK susceptibility in pDST. This finding is consistent with the WHO catalogue descriptions showing that *eis* –14C>T often produces modest increases in MICs [6]. The catalogue further explains that the phenotypic effect of *eis* –14C>T depends on epistasis with mutations in the *eis* coding region, which may enhance or suppress the resistance phenotype. Importantly, the catalogue recommends interpreting *eis* –14C>T as resistant only in situations where pDST is not available, reflecting a precautionary clinical approach. Our isolate, therefore, represents a typical borderline phenotype for this mutation, rather than a discrepancy between molecular and phenotypic methods.

A probable contributor to the reduced sensitivity that was observed in our dataset, particularly for AMK, CAP, and ETH, is the underlying genetic diversity and lineage-dependent variation in the MTB strains that are circulating in Israel. Resistance to these drugs is driven by a wide spectrum of mutations across multiple genes, including promoter variants, coding-region loss-of-function mutations, and insertions or deletions, many of which fall outside the limited genomic targets incorporated into GXXDR cartridges. This implies that the performance of GXXDR may differ between regions with different circulating MTB strains.

This study has several limitations that should be considered when interpreting the findings. First, the sample size was relatively small, which reduces the statistical power for estimating drug-specific sensitivity and specificity, particularly for drugs with low resistance prevalence. Second, not all isolates underwent all three susceptibility tests (pDST, WGS, and GXXDR). In several cases, only two test results were available for comparison, limiting the ability to fully resolve discordant results. Third, KAN was not included in our pDST panel, and, therefore, we were unable to evaluate or report the performance of GXXDR for this drug. Additionally, as the laboratory does not consistently receive comprehensive demographic data for all submitted specimens, demographic variables were not included in the analysis. Despite these limitations, the dataset still provides a valuable real-world assessment of assay performance across a diverse set of clinical isolates.

## 4. Materials and Methods

### 4.1. Data Classification and Identification of MTBC

Patients in Israel suspected of having pulmonary TB are required to provide sputum or other biological specimens for culture, while most suspected cases of extra-pulmonary TB undergo a tissue biopsy for laboratory analysis [20]. All cultures processed nationwide are forwarded to the NMRL in the National Public Health Laboratories, Tel Aviv. At the NMRL, sample smears and TB cultures are stained using the Ziehl-Neelsen technique and cultured on Lowenstein-Jensen media according to established protocols [21]. Cultures are also grown using the BACTEC MGIT 960 system (BD, Sparks, MD, USA). Species identification is carried out with a commercial DNA probe assay (Hain Lifesciences, Nehren, Germany). This retrospective study analyzed laboratory-confirmed TB cases in Israel that underwent GXXDR testing. GXXDR tests (Cepheid, Sunnyvale, CA, USA) were performed according manufacture protocol [10], either upon clinician request or at the discretion of the NMRL. Samples with mixed infections were excluded from the analysis.

pDSTs for MTBC strains were conducted at the NMRL. Testing for INH resistance was performed using either the BACTEC MGIT 960 system or the resistance ratio method (Table 1). For second-line drugs: ciprofloxacin (CIP), CAP, ETH, ofloxacin (OFX), and AMK—the resistance ratio method was employed.

Importantly, for the purpose of the article, the category ‘Borderline’ was redefined as sensitive. The critical concentrations used in the current study are based on the WHO guidelines (https://tbonline.groundup.org.za/media/uploads/documents/who-cds-tb-2018.24-eng.pdf, accessed on 15 February 2026).

### 4.2. Whole Genome Sequencing and Bioinformatics

All the sequencing was done in the NMRL. The genomic DNA from inactivated bacterial cultures was extracted according to the manufacturer’s protocol using the Maxwell RSC cultured cells DNA kit and the Maxwell^®^ 16 System (Promega, Fitchburg, WI, USA) [22]. DNA paired-end libraries were prepared using the Illumina Nextera XT DNA Library Preparation Kit according to Illumina protocols (Illumina, San Diego, CA, USA). For sequencing, we utilized the Illumina MiSeq platform using a MiSeq Reagent Kit v2 (500-cycles) (catalogue MS-102-2003) or a MiSeq Reagent Kit v3 (600-cycle), (catalogue MS-102-3003). Illumina sequences are available to the public at the NCBI (BioProject PRGNA1392169) and ENA EBI-NCBL (Study PRJEB106059).

Raw reads fastq files were quality analyzed by FastQC [23]. Kraken2 taxonomic classification was used to identify mixed cultures [24]. Freebayes was used for the identification of genomic variations in MTBC [25]. The genomic variations were compared with the WHO 2023 *Catalogue of mutations in Mycobacterium tuberculosis complex and their association with drug resistance* to identify antibiotic-resistant strains (isoniazid, levofloxacin, moxifloxacin, amikacin, capreomycin, ethionamide).

In order to perform a comparison between phenotypic and genotypic susceptibility test results, we defined the following categories in the WHO catalogue final confidence grading: resistant, ‘associated with resistance’, and ‘associated with resistance-interim’, as well as mutations that were not in the catalogue, which nevertheless comply with expert rules used for the final confidence grading.

### 4.3. Statistical Analysis

For each drug, clinical sensitivity was defined as the proportion of samples classified as resistant by the composite reference standard that were also detected as resistant by the GXXDR assay. Similarly, clinical specificity referred to the proportion of samples that were determined to be susceptible by the composite reference standard and that were likewise identified as susceptible by the GXXDR assay. Confidence intervals were calculated using the Wilson test.

To compare the diagnostic sensitivity between our results and those reported by the manufacturer from retrospective specimens [10], we performed statistical analysis of sensitivity values using Fisher’s exact test. For each antibiotic, 2 × 2 contingency tables were constructed based on the number of true positive and false negative results obtained in our dataset and in the manufacturer’s data. Fisher’s exact test was applied to assess whether the observed differences in sensitivity between the two datasets were statistically significant.

Similarly, the Tango test for paired data was used to compare the diagnostic sensitivity between GXXDR and WGS testing. This method accounts for the paired nature of the samples tested by both methods and evaluates whether the difference in sensitivity between GXXDR and WGS is statistically significant.

## 5. Conclusions

In conclusion, the GXXDR assay demonstrates high accuracy for detecting resistance to drugs driven by well-characterized canonical mutations, such as INH and FQ, supporting its utility as a rapid first-line molecular diagnostic tool. However, its reduced sensitivity for AMK, CAP, and ETH highlights important limitations related to restricted target regions and the genetic diversity of resistance mechanisms, emphasizing the continued need for complementary pDST and WGS to ensure comprehensive and reliable drug resistance detection.

## Figures and Tables

**Table 1:** Drug threshold levels that were used to determine resistance.

a. BACTECe MGITe 960:			
**Drug**	**Concentration (µg/mL)**		
INH	0.1		
b. Resistance ratio method.			
**Drug**	**Borderline (B) µg/mL**	**Resistant = RR4 µg/mL**	**Highly Resistant µg/mL**
INH	0.05	0.1	0.2
CIP	1.6	3.2	6.4
CAP	14	28	56
ETH	10	20	40
OFX	1.25	2.5	5
AMK	7.5	15	30

## Data Availability

The original contributions presented in this study are included in the article and Supplementary Materials. Further inquiries can be directed to the corresponding author.

## Supplementary Materials

The following supporting information can be downloaded at https://www.mdpi.com/article/10.3390/ijms27062597/s1.
